# Reducing acquisition time for MRI-based forensic age estimation

**DOI:** 10.1038/s41598-018-20475-1

**Published:** 2018-02-01

**Authors:** Bernhard Neumayer, Matthias Schloegl, Christian Payer, Thomas Widek, Sebastian Tschauner, Thomas Ehammer, Rudolf Stollberger, Martin Urschler

**Affiliations:** 1 Ludwig Boltzmann Institute for Clinical Forensic Imaging, Universitätsplatz 4, 8010 Graz, Austria; 2 Institute of Medical Engineering, Graz University of Technology, Stremayrgasse 16, 8010 Graz, Austria; 3 Institute of Computer Graphics and Vision, Graz University of Technology, Inffeldgasse 16, 8010 Graz, Austria; 4 Division of Pediatric Radiology, Department of Radiology, Medical University of Graz, Auenbruggerplatz 34, 8036 Graz, Austria; 5 BioTechMed-Graz, Graz, Austria

## Abstract

Radiology-based estimation of a living person’s unknown age has recently attracted increasing attention due to large numbers of undocumented immigrants entering Europe. To avoid the application of X-ray-based imaging techniques, magnetic resonance imaging (MRI) has been suggested as an alternative imaging modality. Unfortunately, MRI requires prolonged acquisition times, which potentially represents an additional stressor for young refugees. To eliminate this shortcoming, we investigated the degree of reduction in acquisition time that still led to reliable age estimates. Two radiologists randomly assessed original images and two sets of retrospectively undersampled data of 15 volunteers (N = 45 data sets) applying an established radiological age estimation method to images of the hand and wrist. Additionally, a neural network-based age estimation method analyzed four sets of further undersampled images from the 15 volunteers (N = 105 data sets). Furthermore, we compared retrospectively undersampled and acquired undersampled data for three volunteers. To assess reliability with increasing degree of undersampling, intra-rater and inter-rater agreement were analyzed computing signed differences and intra-class correlation. While our findings have to be confirmed by a larger prospective study, the results from both radiological and automatic age estimation showed that reliable age estimation was still possible for acquisition times of 15 seconds.

## Introduction

Age estimation in living individuals is important for clinical applications^1–3^ as well as in legal or forensic medicine investigations^4^ and sports^5–8^, but it is prone to uncertainty caused by the variation of human development^9^. Concerning biological age one can draw insights from comprehensive studies^10,11^ but the use of biological development for estimating chronological age, as required in forensic applications, is still a topic of current research^12,13^. Recently, the increased flow of individuals into and across the European Union raised interest in forensic age estimation for children, adolescents and young adults claiming to be minors but lacking valid identification documents^14^.

Current multi-factorial age estimation methods are based on a radiograph of the hand, a panoramic X-ray image of the teeth and computed tomography images of the clavicles^15^. To avoid exposure to ionizing radiation, there is growing interest in magnetic resonance imaging (MRI) for forensic age estimation^5,16–23^. This interest has led to developments such as a recently proposed fully automatic machine learning based method^24^ based on MR images of the left hand and wrist.

Compared to acquiring radiographs or computed tomography images, MRI has the drawback of considerably longer acquisition times, leading to increased examination costs and reduced patient comfort. Additionally, longer acquisition times are more prone to errors due to motion artefacts when acquiring images of children or adolescents. Therefore, short examination times are highly preferable.

A reduction of MR scanning time can be achieved by leaving out acquisition steps, often termed undersampling. The CAIPIRINHA (Controlled Aliasing In Parallel Imaging Results IN Higher Acceleration) undersampling strategy^25^ enables optimized acceleration for 3D image acquisition and is readily available on current MR scanners. To recover artefact-free images from a reduced amount of data, an advanced reconstruction strategy has to be applied. For this task *total generalized variation* (TGV) regularization^26,27^ has already demonstrated its applicability in various MRI studies^28–31^. We thus anticipate applicability with a high acceleration potential for volumetric MR data for age estimation by using CAIPIRINHA and TGV.

We conduct this feasibility study to investigate the degree of acceleration that can be applied to hand/wrist MRI for age estimation without significantly influencing the estimation outcome. This aims at determining limits and applicability of the proposed method by comparing the reliability of both human and automated evaluation, reflecting the potential of automatic methods to support radiologists in age estimation tasks.

## Methods

### Ethics Statement and Informed Consent

The study was performed in accordance with the Declaration of Helsinki and was approved by the ethical committee of the Medical University of Graz (EK 21–399 ex 09/10). All volunteers provided written informed consent. From underage participants written consent from a legal guardian was additionally obtained.

### Subjects

For this feasibility study 18 healthy male Caucasian volunteers between 13.8 and 23.2 years (mean = 17.2 y, median = 17.0 y) were recruited to acquire three-dimensional MR images of the left hand and wrist. The data of 15 volunteers were used to investigate implications of a reduction in acquisition time on resulting age estimates as described below. The data of the remaining three volunteers (see Table 1) were used to compare retrospectively undersampled images with actually acquired undersampled images.

**Table 1:** Overview of acquisition times and acceleration factors for simulated and acquired data.

Simulated, n = 15 (13.77y–23.15 y, µ = 16.87 y, σ = 2.41)				
Name	AF	t_Acqu_	Radiological analysis	Automatic analysis
I_Orig_	1	226 s	Yes	Yes
I_Sim29_	3.89	29 s	Yes	Yes
I_Sim15_	7.49	15 s	Yes	Yes
I_Sim10_	10.84	10 s	No	Yes
I_Sim8_	13.96	8 s	No	Yes
I_Sim7_	16.86	7 s	No	Yes
I_Sim6_	19.58	6 s	No	Yes
**Acquired, n = 3 (15.75 y, 18.85 y, 21.61 y)**				
I_Acq28_	4.07	28 s	No	No
I_Acq15_	7.55	15 s	No	No
I_Acq8_	13.63	8 s	No	No

µ: mean, σ: standard deviation, AF: acceleration factor describing speed-up of acquisition time, t_Acqu_: acquisition time.

### MR Image Acquisition

MRI exams were performed using commercially available clinical 3 T MR scanners (Skyra/Prisma, Siemens Healthineers, Erlangen, Germany) and a conventional 20-channel receive-only head-neck coil (Siemens Healthineers, Erlangen, Germany). Volunteers were placed in prone position with outstretched left arm. The hand was weighted down using a sandbag to minimize movements.

For all 18 subjects T_1_-weighted 3D FLASH (Fast Low Angle SHot) VIBE (Volumetric Interpolated Breath hold Examination) measurements (T_E_/T_R_/FA = 4.06 ms/14 ms/15°, field-of-view = 129 mm $\times$ 23 0mm, two averages, acquisition matrix = 129 $\times$ 230 and image matrix = 288 $\times$ 512, 72 slices) of the left hand and wrist were acquired. The resulting 3D volumes had an image resolution of 0.45 mm $\times$ 0.45 mm $\times$ 0.90 mm and required an acquisition time of t_Acqu_ = 3:46 minutes. For later comparisons with undersampled data, the images from this fully-sampled data are referred to as *original* images or data.

For three volunteers (see Table 1) *accelerated* measurements were additionally acquired using CAIPIRINHA with 12 calibration lines and acquisition times of 28, 15 and nine seconds.

**Figure 1:**
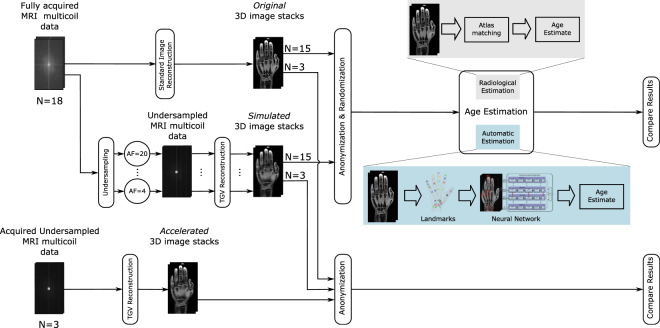
Schematic illustration of the applied method to investigate the reliability of age estimation based on undersampled data. Both original images and images reconstructed from undersampled data (AF: acceleration factor describing speed-up of acquisition time) are used for age estimation applying radiological and automatic estimation methods, respectively. Finally, the differences in the estimates are evaluated. Additionally, simulated data is compared to actually acquired data to show the validity of using retrospectively undersampled data.

For a better understanding, an overview of the study design is given in Fig. 1.

### Retrospective Undersampling of MRI Data

Undersampling MRI raw data is equivalent to not acquiring part of the data, i.e. leaving out data lines during the acquisition. Therefore, retrospectively undersampling conventionally acquired data by removing data lines from the fully-sampled data set *prior to image reconstruction* is a valid reference method to determine specific acceleration potential. The retrospective undersampling of the raw MR data was applied by simulating the commercially available CAIPIRINHA acquisition strategy with minimized noise amplification^32^.

For 15 volunteers, the CAIPIRINHA method with 12 calibration lines was applied retrospectively to simulate six different reduced acquisition times (t_Acqu_) between 29 and six seconds (see Table 1) providing a total of 105 data sets. Only non-averaged data were undersampled, which additionally reduced the required acquisition time by a factor of two, compared to the standard setting of performing two averages. In order to reduce the computational burden, the multi-channel data were reduced to a lower number of virtual coils via coil compression^33^. The virtual coil sensitivities were then estimated from the calibration data with the ESPIRiT method^34^. Image reconstruction was carried out for all simulated acceleration factors (AF) using the TGV method^27^, which considers smooth tissue variations and uses a dedicated optimization algorithm^35^ adapted for parallel computing. When comparing images reconstructed from retrospectively undersampled data to original images they will be referred to as *simulated* images or data.

For the remaining three volunteers, the undersampling patterns were matched exactly to the pattern of the additionally acquired accelerated measurements, simulating acquisition times of 28, 15 and eight seconds, respectively.

The software for image reconstruction is provided online at https://github.com/IMTtugraz/AVIONIC.

### Comparison of Simulated and Acquired Data

For three volunteers (see Table 1), we compared acquired undersampled images with the corresponding simulated images. A comparison of changes of specific image features with increasing undersampling factor in both acquired and simulated data serves the purpose of showing the validity of using retrospectively undersampled data for this study.

### Skeletal Rating

Skeletal age was rated independently using two different methods. A radiologist with more than five years of expertise in forensic applications (R1) and a pediatric radiologist with five years of experience in bone age estimation (R2) independently evaluated whether the quality of the simulated images was adequate for reproducible radiological age estimation. For MRI-based radiological age estimation, radiologists applied the method proposed by Greulich and Pyle^36^ (GP) to the MR images evaluated as assessable. The GP method, originally developed for age estimation based on radiographs, was verified to be applicable for age estimation from MR images, reporting errors on the same scale as inter-rater variations^37^. To avoid biased age estimates the MR images were anonymized and randomized irrespective of the acceleration factor.

To estimate general limits of radiological assessability, an initial analysis was performed after acquisitions of the first five volunteers. The acquired MR data were undersampled according to the values in Table 1. A radiological evaluation rated four out of five data sets with acquisition times below 15 seconds as unusable for a non-ambiguous radiological age estimation. Therefore, for radiological evaluation only original data and simulated image stacks with acquisition times of 29 and 15 seconds – a total of 45 data sets – were presented to radiologists R1 and R2 for age estimation.

The second skeletal age rating was performed using the fully automated age estimation method proposed by Urschler *et al*.^24^ extended by improving landmark localization accuracy^38^ and introducing a novel deep neural network based age estimator^39^. This setup was used solely as an age predictor, i.e. without using data from the present study to further train the model or tune its parameters.

### Statistical Analysis

The main focus of this study was on the reliability of age estimation with decreasing acquisition time and not on the actual absolute results of age estimation. Therefore, we analyzed the difference introduced into the estimated age with decreasing acquisition time to assess reliability. For this analysis the reference age for comparison was the age estimated by each observer using the original images. The difference was then calculated by subtracting the age estimated from original data from the age estimated from simulated data for results of radiologist R1 and R2 (${\Delta {\rm{Age}}}_{R1}$, ${\Delta {\rm{Age}}}_{R2}$) and the automatic age estimation (${\Delta {\rm{Age}}}_{{autom}}$):

$${\Delta }{{\rm{Age}}}_{R1}={{\rm{Age}}}_{R1}-{{\rm{Age}}}_{R1,orig}$$

$${\Delta }Ag{e}_{R2}=Ag{e}_{R2}-Ag{e}_{R2,orig}$$

$${\Delta }{{\rm{Age}}}_{autom}={{\rm{Age}}}_{autom}-{{\rm{Age}}}_{autom,orig}$$

The standard deviation of the signed differences (SSD) of $\Delta {\rm{Age}}$ was used as a measure for the reliability of the age estimation, the mean of signed differences (MSD) to identify potential systematic errors. Additionally, the intra-class correlation coefficient *ICC* was calculated between the age estimates based on original images and the estimates from simulated data sets.

The inter-rater reproducibility between all three observers, i.e. R1, R2 and the automatic age estimation method (A), was determined by calculating ICC and Bland-Altman limits of agreement (LOA) between corresponding age estimates. The inter-rater reproducibility between the radiological estimation and the automatic method thereby provides a measure of conformity between the two different age estimation methods. This information may help to evaluate the potential to combine them to a hybrid between manual and fully automatic age estimation similar to an approach recently proposed for volumetry in oncology^40^.

All statistical analyses were performed using MATLAB (R2014b, The MathWorks Inc., Natick, MA, USA).

### Data Availability

The acquired MRI data sets generated and/or analyzed during the current study are not publicly available for data privacy reasons. The participants did not explicitly give their consent to freely distribute their imaging data, albeit anonymized. However, quantitative measures derived from the imaging data will be made available as a supplementary to this publication.

### Ethical approval

All procedures performed in studies involving human participants were in accordance with the ethical standards of the institutional and/or national research committee and with the 1964 Helsinki declaration and its later amendments or comparable ethical standards.

## Results

### Image Reconstruction and Image Quality

**Figure 2:**
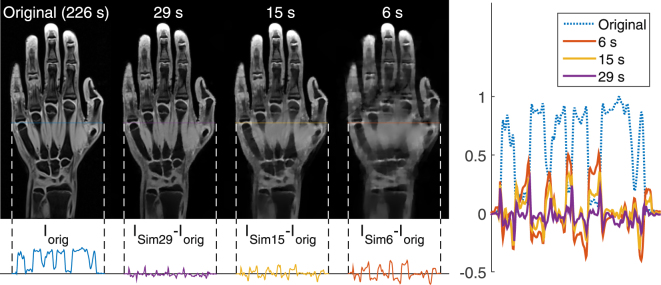
Exemplary images of a selected slice of one volunteer (14.2 y) for originally acquired data, I_orig_, and simulated images I_Sim29_, I_Sim15_ and I_Sim6_. Differences between original and reconstructed images are additionally displayed for selected image profiles.

Figure 2 shows representative images of a central slice of the left hand and wrist of one volunteer (14.2 y) for the original data set and simulated acquisition times of 29, 15 and six seconds. Qualitatively, for an acquisition time of at least 15 seconds no severe artefacts can be identified; however, images with an acquisition time of 15 seconds already feature image blurring, which increases with the acceleration factor. For an acquisition time of six seconds, differences between original and simulated data become clearly visible.

Additionally, the difference between original and simulated images is shown for an image profile line covering bone, muscle tissue and joint cartilage. Deviations from the original image increase with the acceleration factor and become pronounced for larger muscle regions. In general, the reduction of available data leads to blurring and loss of morphological details in the resulting images. This blurring is observable for example as overlap of the muscle tissue with metacarpal bones or the broadening of the joint cartilage of the fifth digit (first visible for t_Acqu_ = 15 s) producing positive peaks in the difference of the profile lines.

### Assessability of Simulated MR Images

All images with acquisition times of 29 and 15 seconds were rated as suitable for age estimation by both radiologists. The automatic age estimation provided age estimates for all data sets.

### Variability and Reliability of Rating

**Figure 3:**
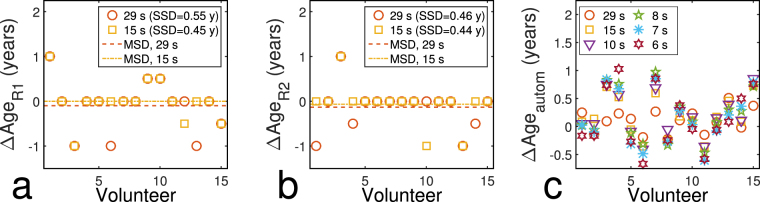
Differences to age estimates based on original data set introduced by a reduction of the acquisition time. Differences are shown for (**a**) R1, (**b**) R2 and (**c**) the automatic age estimation method as a function of the acquisition time. Lines in (**a**) and (**b**) mark the MSD value for each acceleration factor (exact values are shown in Table 2).

Figure 3 visualizes the influence of the reduction of acquisition time on age estimation by showing the difference to the age estimates based on the original data for radiologists R1 and R2 and the automatic method (the values for all age estimates can be found in Supplementary Table S1 online). For the radiological evaluation, standard deviations of signed differences (SSD) introduced by simulated acceleration were 0.57 y and 0.46 y for acquisition times of 29 and 15 seconds, respectively, for R1 and 0.46 y and 0.44 y for R2; the corresponding values for the mean deviations (MSD) were −0.10 y and 0.00 y for R1 and −0.13 y and −0.07 y for R2. For automatic age estimation, SSD values increased with the acceleration factor and reached a maximum of 0.51 y for t_Acqu_ =6 s, MSD values lay between 0.10 and 0.21 years; all SSD and MSD values are provided in Table 2.

**Table 2:** Comparison between ratings of radiological and automatic age estimation: reliability of age estimates is reported as correlation with estimates based on fully-sampled data sets.

Name	R1			R2			Automatic Analysis		
	ICC	SSD (y)	MSD (y)	ICC	SSD (y)	MSD (y)	ICC	SSD (y)	MSD (y)
I_Sim29_	0.96	0.55	−0.1	0.97	0.46	−0.13	0.99	0.21	0.10
I_Sim15_	0.98	0.45	0	0.98	0.44	−0.07	0.98	0.34	0.18
I_Sim10_	—	—	—	—	—	—	0.98	0.37	0.21
I_Sim8_	—	—	—	—	—	—	0.97	0.43	0.21
I_Sim7_	—	—	—	—	—	—	0.97	0.46	0.15
I_Sim6_	—	—	—	—	—	—	0.96	0.51	0.14

ICC: Intra-class correlation coefficient, SSD/MSD: Standard deviation/mean of signed differences.

**Figure 4:**
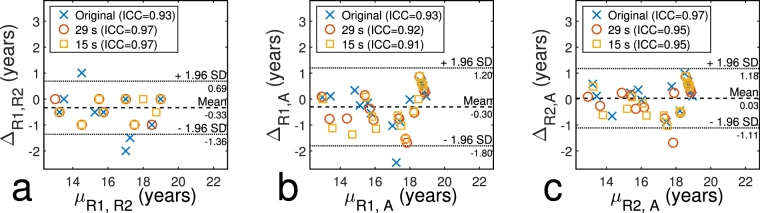
Bland-Altman plots for inter-rater agreement. Agreement is shown between (**a**) R1 and R2, (**b**) R1 and the automatic method (A) and (**c**) R2 and the automatic method as a function of the acquisition time. µ_R1,R2_, µ_R1,A_ and µ_R2,A_, describe the mean value of the age estimates of the respective raters, Δ is the difference between the respective ratings.

The values for the ICC in Table 2 show high intra-class correlation for both applied age estimation methods. A comparison to original age estimates yields a minimum ICC of 0.96 for all evaluated data sets; the values for inter-rater variability lay between 0.91 and 0.99. All results were highly significant with p < 0.000001 for all values. The Bland-Altman plots in Fig. 4 show high inter-rater agreement. The mean values of the Bland-Altman analysis lie between 0.03 and 0.33 years and suggest no systematic error in the analysis. Radiological raters R1 and R2 show the best agreement with LOA = 1.02 y, testing the agreement between radiological and automatic method yields LOA = 1.5 y for R1 and LOA = 1.14 y for R2.

The automatic method as well as both radiologists estimated the oldest volunteer (23.2 y) to be over 18 y for all acceleration factors. The evaluation by the radiologists yielded 19 y for all acceleration factors for this volunteer – the maximally assessable age – and the automatic estimation provided estimates between 18.3 y and 18.9 y.

### Comparing Simulated and Acquired Data

**Figure 5:**
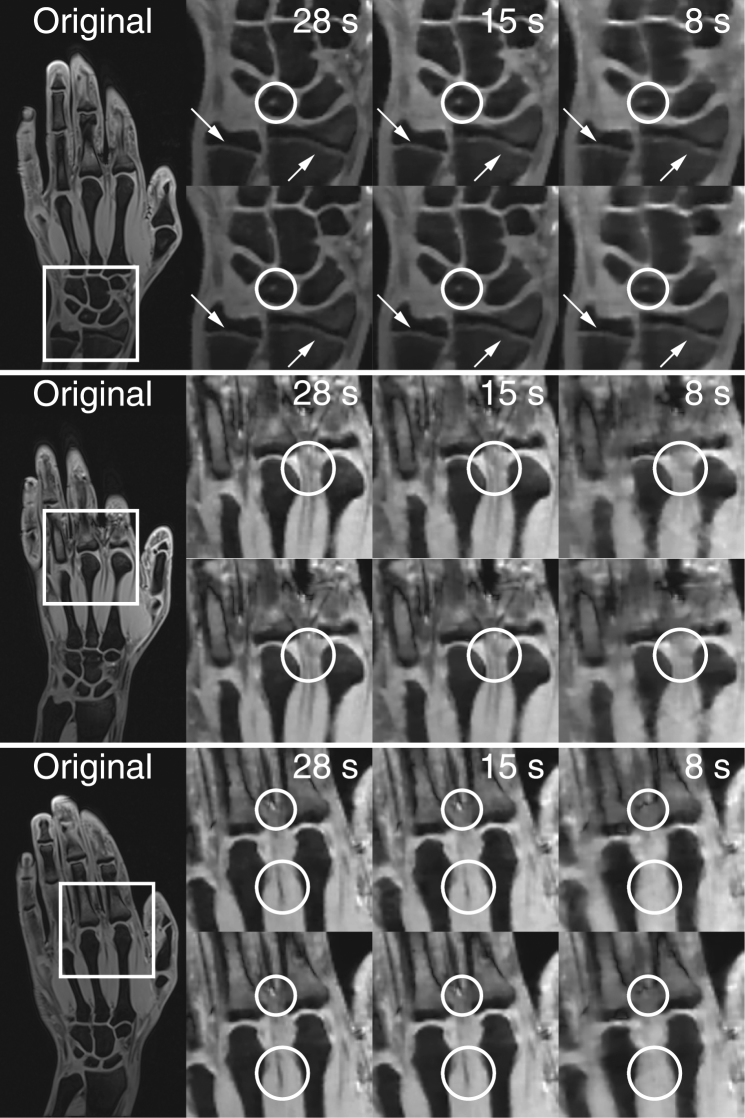
Comparison of simulated (upper rows) and acquired (lower rows) undersampled data for three different volunteers (15.75, 18.85 and 21.61 years from top to bottom) and locations. Arrows mark structures relevant for age estimation, while circles highlight structures changing their appearance with decreasing acquisition time in both simulated and acquired data.

Figure 5 compares simulated data with actually acquired undersampled data of three volunteers (15.75, 18.85 and 21.61 years from top to bottom) showing image details for simulated (upper rows) and acquired (lower rows) accelerated MRI and acquisition times of 29, 15 and eight seconds. For the youngest volunteer epiphyseal gaps are still visible for an acquisition time of eight seconds and the hyperintense structure marked by circles is blurred equally for simulated and acquired images with decreasing acquisition time. Structures marked in the images of the remaining two volunteers become noticeably blurred for an acquisition time of 15 seconds and disappear for further acceleration; again, this behavior can be seen in both simulated and acquired data.

## Discussion

The presented results suggest that a radiological analysis can provide reliable age estimates based on hand/wrist MRI using an acquisition time of only 15 seconds, which corresponds to an acceleration factor of approximately 7.5 compared to the original acquisition time of 3:46 minutes. For this duration no relevant artefacts occurred in the simulated images and all data sets were deemed assessable and yielded a maximum SSD of 0.55 years (shown in Fig. 3). This is in the range of reported errors for the radiological examination of skeletal development^41^. With decreasing acquisition time, automatic age estimation showed an increasing deviation compared to the estimation from the original data set. However, for a simulated duration of six seconds the standard deviation was still only 0.51 years (see Table 2). Both estimation methods yielded small MSD values suggesting that age estimates are not influenced by a systematic offset.

With increasing acceleration factor, images reconstructed from undersampled data tend to appear blurry while noise is suppressed and fine structures become less distinctive, creating an unusual image representation for radiologists. The quality of the simulated images allowed a radiological analysis for acquisition times down to 15 seconds and age estimates for the analyzed data sets were close to identical. The automatic method provided reliable results even for the shortest acquisition time of six seconds. This is a remarkable reduction of the acquisition time as existing age estimation studies at a field strength of 3 Tesla can require acquisition times of up to six minutes for the wrist only^7^. The potential acceleration is markedly higher than acceleration factors reported in a recent study by Terada *et al*. reducing the acquisition time by a factor of 4 from 2:44 minutes to 41 seconds^42^. However, our results cannot easily be compared to the work of Terada *et al*., since they used a low-field MR scanner at 0.3 Tesla. A lower field strength generally bears the disadvantage of lower SNR but also allows shorter acquisition times due to shorter T_1_ relaxation times. Above that, Terada *et al*. applied an optimized undersampling pattern for their compressed sensing-based approach, which is not commercially available.

The comparison of standard deviations of radiological and automatic analysis methods has to be interpreted carefully, since the minimal deviation that may occur using the GP atlas-matching scheme is 0.5 years, while the automatic method provides a continuous age estimate. Furthermore, contrary to the modern-day reference population of the automatic method, the GP scheme uses a different reference population consisting of Caucasian volunteers born in the 1930’s, which may be considered outdated due to changes in multinational behavior. From a methodological point of view, the difference between the acceptable acceleration factor for an analysis by a radiologist and that for the automatic method could be explained by the fact that the automatic age estimation algorithm analyses the entire 3D data set simultaneously. This avoids influences of single artefacts mimicking a partial closure of the epiphyseal gap in a 2D representation.

The main aim of this study was to test reliability. However, the oldest volunteer (23.15 years) was included to test whether image reconstruction may introduce misleading image features causing an estimation of under 18 years – a legally important age threshold indicating majority age in many countries. Based on the atlas, the maximum age a radiologist can allocate is 19 years old. In the oldest volunteer this maximum age was allocated to image stacks of all acceleration factors. Accordingly, the automatic estimation also provided estimates over 18 y for all acceleration factors, which suggests that the chosen undersampling and reconstruction strategies are robust against misleading artefacts for the simulated acceleration factors.

The validity to use retrospectively undersampled data in this study was shown by a comparison of simulated and acquired images. The simulation of an accelerated acquisition removes data lines that are not acquired during an actual acquisition. Therefore, an agreement between simulated and acquired data can be anticipated. Even more, retrospective undersampling represents a worst-case simulation since the reduced amount of data is extracted from a long acquisition during which more patient movement can occur. For this reason the additional acquisition of accelerated data sets was only performed for a small number of volunteers.

Our work is based on an undersampling scheme readily available on current MR scanners and therefore does not require comprehensive knowledge on undersampling strategies or MR sequence programming. The same applies to the image reconstruction algorithm, which is available to the public in an online repository. This allows for an easy adoption of our proposed method. It is also noteworthy that the automatic method did not require additional training and could readily be applied to undersampled data in its original state. Using the concept of systematically increasing the undersampling of the available data, the feasibility of our approach could already be shown for the relatively small sample size used in this study. We expect to reproduce these results with more data sets, which are currently being acquired.

The potential decrease in acquisition time presented in this study is an important step towards establishing MRI as standard method for age estimation. On successful transfer of this approach to MR acquisitions of third molars and clavicular epiphyses, the application of MRI for multi-factorial age estimation could be promoted even further due to the elimination of the drawback of time consumption. Furthermore, this also translates to a potential reduction of the cost of using this ionizing radiation-free imaging modality for age estimation.

In conclusion, we showed the reliability of image data undersampled with the CAIPIRINHA technique in combination with TGV-based reconstruction for skeletal age estimation. A reduction of the acquisition time to 15 seconds for MR acquisitions of the hand and wrist was found to produce images interpretable using both a radiological and an automatic age estimation method. Furthermore, the high correlation between the two methods shows the potential of automatic methods to support radiologists in age estimation investigations.

## Electronic supplementary material

Supplementary Table S1

## References

[1] Martin, D. D. *et al*. The use of bone age in clinical practice–part 1. *Horm Res Paediatr* **76**, 1–9 (2011). 10.1159/000329372 21691054

[2] Lee SC, Shim JS, Seo SW, Lim KS, Ko KR (2013). The accuracy of current methods in determining the timing of epiphysiodesis. Bone Jt. J.

[3] Wang, W. W. J. *et al*. Correlation of Risser sign, radiographs of hand and wrist with the histological grade of iliac crest apophysis in girls with adolescent idiopathic scoliosis. *Spine* (*Phila Pa 1976*) **34**, 1849–1854 (2009). 10.1097/BRS.0b013e3181ab358c 19644336

[4] Schmeling A, Geserick G, Reisinger W, Olze A (2007). Age estimation. Forensic Sci Intl.

[5] Dvorak J, George J, Junge A, Hodler J (2007). Age determination by magnetic resonance imaging of the wrist in adolescent male football players. *Br J Sport*. Med.

[6] George J, Nagendran J, Azmi K (2012). Comparison study of growth plate fusion using MRI versus plain radiographs as used in age determination for exclusion of overaged football players. *Br J Sport*. Med.

[7] Schmidt S, Vieth V, Timme M, Dvorak J, Schmeling A (2015). Examination of ossification of the distal radial epiphysis using magnetic resonance imaging. New insights for age estimation in young footballers in FIFA tournaments. Sci. Justice.

[8] Timme M, Steinacker JM, Schmeling A (2017). Age estimation in competitive sports. Int. J. Legal Med..

[9] Cameron N (2015). Can maturity indicators be used to estimate chronological age in children?. Ann Hum Biol.

[10] Tanner, J. M. *A history of the study of human growth*. (Cambridge University Press, 1981).

[11] Ulijaszek, S. J., Johnston, F. E. & Preece, M. A. *The Cambridge encyclopedia of human growth and development*. (Cambridge University Press, 1998).

[12] Liversidge HM, Buckberry J, Marquez-Grant N (2015). Age estimation. Ann Hum Biol.

[13] van Rijn R, Thodberg H (2013). Bone age assessment: automated techniques coming of age?. Acta radiol..

[14] Schmeling, A., Garamendi, M. P., Prieto, J. L. & Landa, I. M. Forensic Age Estimation in Unaccompanied Minors and Young Living Adults. *Forensic Med*. *- From Old Probl*. *to New Challenges*10.5772/19261 (2011).

[15] Schmeling, A. *et al*. Updated recommendations of the Study Group on Forensic Age Diagnostics for age estimation in the living in criminal proceedings. *Rechtsmedizin* **18**, 451–453 (2008).

[16] Hillewig E (2011). Magnetic resonance imaging of the medial extremity of the clavicle in forensic bone age determination: a new four-minute approach. Eur. Radiol..

[17] Dedouit F (2012). Age assessment by magnetic resonance imaging of the knee: A preliminary study. Forensic Sci Intl.

[18] Terada Y (2013). Skeletal age assessment in children using an open compact MRI system. Magn Reson Med.

[19] Tomei E (2013). Value of MRI of the hand and the wrist in evaluation of bone age: Preliminary results. J Magn Reson Imaging.

[20] Serinelli S (2015). Accuracy of MRI skeletal age estimation for subjects 12–19. Potential use for subjects of unknown age. Int J Leg. Med.

[21] Baumann P (2015). Dental age estimation of living persons: Comparison of MRI with OPG. Forensic Sci Intl.

[22] De Tobel J, Hillewig E, Verstraete K (2017). Forensic age estimation based on magnetic resonance imaging of third molars: converting 2D staging into 3D staging. Ann. Hum. Biol..

[23] Ekizoglu O (2016). Forensic age estimation via 3-T magnetic resonance imaging of ossification of the proximal tibial and distal femoral epiphyses: Use of a T2-weighted fast spin-echo technique. Forensic Sci. Int..

[24] Urschler M, Grassegger S, Štern D (2015). What automated age estimation of hand and wrist MRI data tells us about skeletal maturation in male adolescents. Ann Hum Biol.

[25] Breuer FA (2005). Controlled aliasing in parallel imaging results in higher acceleration (CAIPIRINHA) for multi-slice imaging. Magn Reson Med.

[26] Bredies K, Kunisch K, Pock T (2010). Total generalized variation. SIAM J Imaging Sci.

[27] Knoll F, Bredies K, Pock T, Stollberger R (2010). Second order total generalized variation (TGV) for MRI. Magn Reson Med.

[28] Knoll F, Clason C, Bredies K, Uecker M, Stollberger R (2012). Parallel Imaging with Nonlinear Reconstruction using Variational Penalties. Magn Reson Med.

[29] Knoll F (2013). Reconstruction of undersampled radial PatLoc imaging using total generalized variation. Magn Reson Med.

[30] Valkonen T, Bredies K, Knoll F (2013). Total Generalized Variation in Diffusion Tensor Imaging. SIAM J Imaging Sci.

[31] Langkammer C (2015). Fast quantitative susceptibility mapping using 3D EPI and total generalized variation. Neuroimage.

[32] Athalye V, Lustig M, Uecker M (2015). Parallel magnetic resonance imaging as approximation in a reproducing kernel Hilbert space. Inverse Probl.

[33] Buehrer M, Pruessmann KP, Boesiger P, Kozerke S (2007). Array compression for MRI with large coil arrays. Magn Reson Med.

[34] Uecker M (2014). ESPIRiT–an eigenvalue approach to autocalibrating parallel MRI: Where SENSE meets GRAPPA. Magn Reson Med.

[35] Chambolle A, Pock T (2011). A First-Order Primal-Dual Algorithm for Convex Problems with Applications to Imaging. J Math Imaging Vis.

[36] Greulich WW, Pyle SI (1959). Radiographic atlas of skeletal development of the hand and wrist. Am J Med Sci.

[37] Urschler M (2016). Applicability of Greulich–Pyle and Tanner–Whitehouse grading methods to MRI when assessing hand bone age in forensic age estimation: A pilot study. Forensic Sci Intl.

[38] Payer, C., Štern, D., Bischof, H. & Urschler, M. Regressing Heatmaps for Multiple Landmark Localization Using CNNs. *Med*. *Image Comput*. *Comput*. *Interv*.*–MICCAI 2016: 19th International Conference*, *Athens*, *Greece*, *October 17–21*, *2016*, *Proceedings*, *Part II*, 230–238 (Springer International Publishing, 2016).

[39] Štern, D., Payer, C., Lepetit, V. & Urschler, M. Automated Age Estimation from Hand MRI Volumes Using Deep Learning. *Med*. *Image Comput*. *Comput*. *Interv*.*–MICCAI 2016 19th Int*. *Conf*. *Athens*, *Greece*, *Oct*. *17–21*, *2016*, *Proceedings*, *Part II*, 194–202 (2016).

[40] Kleesiek J (2016). Virtual Raters for Reproducible and Objective Assessments in Radiology. Sci. Rep..

[41] Ritz-Timme S (2000). Age estimation: The state of the art in relation to the specific demands of forensic practise. Int J Leg. Med.

[42] Terada Y (2015). Acceleration of skeletal age MR examination using compressed sensing. J. Magn. Reson. Imaging.

