# Supplementary material for: Reducing acquisition time for MRI-based forensic age estimation
Source: Sci Rep. 2018 Feb 1;8:2063. doi: 10.1038/s41598-018-20475-1 (PMC5794919; doi:10.1038/s41598-018-20475-1)
Supplement: Supplementary file 1 — Supplementary Table S1 [file 41598_2018_20475_MOESM1_ESM.pdf]

Reducing acquisition time for MRI-based forensic age estimation

Bernhard Neumayer, MSc, Matthias Schlögl, MSc, Christian Payer, MSc, Thomas Widek, MSc, Sebastian Tschauner, MD, Thomas Ehammer, MD, Rudolf Stollberger, PhD, Martin Urschler, PhD

Volunteer #	Orig years	29 seconds years	15 seconds years
1292	16.00	17.00	17.00
1293	17.00	17.00	17.00
1296	15.00	14.00	14.00
1297	15.50	15.50	15.50
1299	19.00	19.00	19.00
1300	19.00	18.00	19.00
1301	15.50	15.50	15.50
1307	19.00	19.00	19.00
1308	15.00	15.50	15.50
1309	16.50	17.00	17.00
1312	19.00	19.00	19.00
1314	19.00	19.00	18.50
1315	18.00	17.00	18.00
1317	13.00	13.00	13.00
1318	13.50	13.00	13.00

Table 1: Age estimates by radiologist R1

Volunteer #	Orig years	29 seconds years	15 seconds years
1292	18.00	17.00	18.00
1293	17.00	17.00	17.00
1296	14.00	15.00	15.00
1297	16.00	15.50	16.00
1299	19.00	19.00	19.00
1300	19.00	19.00	19.00
1301	16.00	16.00	16.00
1307	19.00	19.00	19.00
1308	15.50	15.50	15.50
1309	18.00	18.00	17.00
1312	19.00	19.00	19.00
1314	19.00	19.00	19.00
1315	19.00	18.00	18.00
1317	13.50	13.00	13.50
1318	13.50	13.50	13.50

Table 2: Age estimates by radiologist R2

Volunteer #	Orig years	29 seconds years	15 seconds years	10 seconds years	8 seconds years	7 seconds years	6 seconds years
1292	18.44	18.69	18.52	18.49	18.46	18.46	18.28
1293	17.88	17.74	18.02	17.93	17.82	17.78	17.72
1296	14.66	14.75	15.37	15.41	15.49	15.49	15.40
1297	15.65	15.88	16.17	16.21	16.41	16.32	16.67
1299	18.50	18.64	18.44	18.40	18.39	18.18	18.23
1300	18.72	18.52	18.36	18.30	18.40	18.23	18.05
1301	16.04	16.31	16.64	16.74	17.02	16.89	16.90
1307	18.37	18.14	18.13	18.31	18.04	18.10	18.13
1308	15.25	15.36	15.43	15.53	15.60	15.53	15.63
1309	17.52	17.76	17.64	17.70	17.61	17.55	17.48
1312	18.89	18.75	18.54	18.54	18.42	18.29	18.31
1314	18.34	18.41	18.51	18.49	18.41	18.26	18.27
1315	18.02	18.53	18.47	18.42	18.32	18.23	18.10
1317	12.93	12.90	13.01	13.04	13.20	13.29	13.42
1318	13.40	13.77	14.12	14.25	14.11	14.19	14.15

Table 3: Age estimates by automatic age estimation
